# Implications of COVID-19 infections in sickle cell disease

**DOI:** 10.11604/pamj.2020.36.81.23776

**Published:** 2020-06-09

**Authors:** Nitin Ashok John, Jyoti Elgiva John

**Affiliations:** 1 Department of Physiology, Dr Ram Manohar Lohia Institute of Medical Sciences, Lucknow, India,; 2 Department of Biochemistry, All India Institute of Medical Sciences, Nagpur, India

**Keywords:** Sickle cell disease, Africa, COVID-19, pulmonary, mortality, global, advisory

## Abstract

Sickle cell disease is a major concern of public health significance in Africa. Nearly 2/3^rd^ of the global burden of sickle cell disease (SCD) is found to be in sub-Saharan Africa. There is increased mortality risk in sickle cell disease patients in Africa due to associated complications such as acute chest syndrome, asthma, pulmonary emboli and sepsis. Sickle cell disease management is the major contributor of financial burden on the government. Moreover, there is a shortage of medical specialists in Africa. COVID-19 pandemic has further led to devastating impact on economy and health globally. The chances of SCD patient contracting COVID-19 infections are higher as these patients are immunocompromised and may be at a higher risk of mortality. Practicing preventive measures including isolation and social distancing by these patients will prevent mortality rates as well as economic burden on government in the present unprecedented COVID-19 pandemic.

## Commentary

World Health Organization has identified sickle cell disease (SCD) as a major concern of public health significance. It has been estimated that around 5% of the global population carry sickle cell trait genes. Around 2/3^rd^ of the sickle cell disease patients of the global burden reside in sub-Saharan Africa [1]. SCD has been a neglected cause of childhood mortality in African countries. In view of the huge population of SCD patients in Africa, the government has to bear large financial burden for management of this haemoglobinopathy. Governments in Africa have to struggle while addressing health concerns in SCD by regularly screening all newborns for HbS, providing hydroxyurea therapy and prophylactic medication for pneumococcal infections as public health measures. The perpetual problem of acute shortage of medical specialist across the region adds to further woes of the governments [2]. COVID-19 pandemic caused by corona virus 2 (SARS COV2) is having a devastating effect on socioeconomic and health indicators in counties worldwide as well as in Africa. The additional financial burden of supporting health care management system in tackling COVID-19 impact at the same time preventing mortality rate of COVID-19 deaths is a matter of great concern in Africa [3].

**Pathophysiology of sickle cell disease:** the pathogenesis of the sickle cell disease is attributed to the polymerization of the deoxygenated HbS. The polymerization leads to alteration in the normal biconcave shape of the red blood cells making them rigid and more prone for intravascular haemolysis. As a consequence of repeated hypoxia driven polymerization of HbS there is development of cyclic cascade leading to blood cell adhesion, vaso-occlusive crisis and ischemic reperfusion injury. SCD patients may develop complications such as acute chest syndrome, pulmonary embolism and stroke [4].

**SCD in Africa:** the number of newborns born with SCD worldwide as estimated in 2016 was 3, 12,000. Around 2/3^rd^ of these children are found to be born in sub-Saharan Africa. The sickle cell gene HbSS is commonly identified in Africa in SCD while HbSC and HbS/beta+thalassemia has been observed in West Africa [5], SCD has been a neglected disease in Africa for many years and had led to the death of about 50-90% of the affected as the disease remains undiagnosed during the childhood. The various studies done in Africa were found that SCD patients have higher mortality rates [1, 5]. The development of knowledge of understanding the pathology and management protocol of SCD has been helpful in management of disease but application of these protocols in Africa are limited due to shortage of trained medical personnel and health care facilities such as indoor hospitals, medications and diagnostic facility for newborns HbS screening [2]. The presence of malaria, undernutrition and other infectious diseases are also contributors towards mortality rate in Africa. Of late, it has been seen that because of the devoted and dedicated health care services provided by the health personnel, the mortality rates are declining and this life-threatening disease of children is now progressing to chronic disease of the adult. The study carried by researchers in Kilfi area of Kenya compared the incidence of specific clinical outcomes in children with and without sickle cell disease in age groups between newborn to 5 years. They found that though morbidity and mortality were higher in children with sickle cell disease, these were reduced by early diagnosis and supportive care management. The authors recommended that early detection will help preventing long term complications [6]. Still, till date genetic and infectious diseases in Africa are of public health significance and needs to be addressed by adequate government funding for their effective management.

**Pulmonary complications in sickle cell disease:** it has been observed that pulmonary functions are decreased in SCD. In our previous study also, we found that lung functions were compromised in patients of sickle cell disease and sickle cell trait (SCT). Our finding was attributed to the fact that repeated chest infections in SCD and SCT leads to alteration in geometry of lung parenchyma and physical properties of elastic and collagen fibres thus decreasing pulmonary function parameters such as forced vital capacity, forced expiratory volume and forced expiratory volume 1%. Moreover, the pulmonary vasculature is highly sensitive to hypoxia driven micro-occlusion of pulmonary vasculature which along with cell adhesive changes may cause pulmonary hypertension and further compromise lung functions [4]. The research studies have pointed out that patients with SCD have an increased susceptibility to infection. The impaired leucocyte function and humoral and cell-mediated immunity loss have been reported to account for the immunocompromised state in patients with sickle cell disease [7]. The SCD patients being immune compromised are more prone for recurrent chest infections. The major cause of mortality in patients of SCD is acute chest syndrome, pneumonia and acute respiratory distress syndrome [5].

**COVID-19 pandemic and health impact:** COVID-19 is the acronym for corona virus disease 19 and has been termed as SARS-COV-2 by International Committee of Taxonomy on Virus (ICTV). The common clinical manifestations observed in patients of sickle cell disease include cough, fever, shortness of breath, loss of smell perception and loss of taste sensation. Most of the patients of COVID-19 may have a mild course of disease while few may develop severe clinical manifestations. The clinical manifestation of severity in COVID-19 patient includes acute respiratory distress syndrome (ARDS), pneumonia, multiple organ failure, septic shock and sepsis. The severity of pneumonia manifests with dyspnoea and tachyponea [8, 9].

**COVID-19 and sickle cell disease:** COVID-19 infection can exacerbate the pulmonary manifestation in SCD patients especially in those having pulmonary complications such as acute chest syndrome, pulmonary hypertension and ARDS. COVID-19 infections in SCD can also increase morbidity and mortality risk in these patients [8, 9]. Faiz A *et al*. presented a case series of four sickle cell disease patients who were diagnosed as COVID-19 positive cases and had past history of pulmonary complications such as acute chest syndrome, asthma or pulmonary embolism and they were of the opinion that there is identified potential risk factor for COVID-19 pulmonary disease in these patients. Though these four patients had mild clinical course of COVID-19 the authors advocated further studies with larger sample size. The authors also pointed out that COVID-19 infection disproportionately affects more African American than other ancestry [9].

The main cause of concern in patients of SCD is that these patients are immunocompromised and may suffer from both acute and chronic complications which require hospitalization and close contact with the medical system. There is overlap in clinical manifestations of fever and lung disease in COVID-19 and SCD. The increased complications will amplify health care utilization. The shortage of medical specialists catering towards health care will further hamper and hinder the diagnostic, management and logistic challenges in Africa. In view of the above facts it is necessary for health care workers to educate SCD patients registered in their areas regarding care and precautions to be taken during COVID-19 pandemic to prevent getting affected with COVID-19 infection [8, 10].

**Sickle cell disease advisory by sickle cell disease association of Africa for sub-Saharan Africa** [10]**:** advisory for patients of SCD regarding COVID-19: all patients of SCD need to be educated regarding COVID-19 signs, symptoms and mode of spread. They should be explained regarding the increased risk of contracting COVID-19 infections in them due to their immunocompromised state. All patients of SCD should be advised to strictly adhere to social distancing, isolation polices, use of face mask, and frequent hand washing with soap to prevent COVID-19 infections. They should keep adequate medication of SCD such as analgesic and antipyretic drugs, hydroxyurea and other chronic medication such as L-glutamine, Voxelotor and Crizalizumab etc. depending on drugs which was prescribed to them. This will prevent unnecessary hospital visits and chances of exposure to COVID-19 infections. They can seek consultation with doctors on telephone.

They can be advised regarding use of clinical thermometer at home as fever is common sign in SCD patient and thereby these patients can take appropriate precautions and medication after seeking telephonic consultation with their doctors. They can use pharmacy home delivery services in case they require medication during emergency situations and if their symptoms become severe and they require blood transfusion they should take all precautionary measures when they visit the hospital for emergency care.

**SCD patients reporting for hospital care:** the sickle cell disease advisory by sickle cell disease association of Africa for sub-Saharan Africa has recommended following the standard care of management for SCD patients reporting with suspected COVID-19 infection symptoms such as fever, cough or shortness of breath. They recommended that test for blood culture, COVID-19 testing and prescription of broad spectrum antibiotics should be carried with care and caution and the medical specialist should make a proper assessment for acute chest syndrome [10].

SCD patients being immunocompromised may have greater risk of contracting COVID-19 infection. Due to the pulmonary compromised state of SCD patients especially in SCD with acute chest syndrome or pulmonary hypertension, they are likely to develop fatal complications with COVID-19 infection as fever and respiratory symptoms are the common riders in SCD as well as COVID-19. Thus, SCD patients may be at higher risk of mortality if they get exposed to COVID-19 infection. The precautionary measures of social distancing, isolation, telephonic consultation with doctors if need arises, wearing of face mask and regular washing of hands with soap may help patients of SCD in coping with the COVID pandemic health impact. The well guided preventive measures by doctors and public will also prevent financial burden from piling up in the present unprecedented COVID-19 pandemic. Moreover, further studies regarding impact of COVID-19 in patients of SCD shall further give insight for effective management of these patients.
